# Neuralgia Cured by Galvanism

**Published:** 1834

**Authors:** 


13. *Neuralgia of the face successfully treated by Galvanism, applied in the manner recommended by Mansford.*—Dr. Harris, of Philadelphia, in the periodical just quoted, has given an account of several cases of this kind, drawn from his own practice, of which the following is one:

"In May, 1834, Mr. T. W. of Maryland, consulted me in a case of neuralgia of the head and face of eighteen months continuance. He had been under medical treatment during the whole of this period, but could not state the names or nature of the medicines which he had taken. His pain was very acute; his nights were sleepless; his digestive functions much impaired, with obstinately constipated bowels. His extremities were cold, and his head inordinately hot. A few ounces of blood was taken from the back of his neck, aperients were given to regulate his bowels, and his feet were placed nightly in a mustard foot-bath. On the third day the galvanic apparatus was applied, and was continued two weeks before any mitigation of the pain was observed. At the expiration of five weeks the patient was entirely relieved of his neuralgia, and his health in every particular entirely restored. Within a few days he has returned to his native state in fine health and spirits.

As many of our readers may not possess Mansford's book on Epilepsy, which contains the mode in which he directs the galvanic apparatus to be employed, we shall transcribe it:

"It was said, that in order to fulfil the indication stated at the commencement of this section, it was desirable to establish a negative point as near the brain as possible, and a positive one in some distant part of the body. Accordingly, a portion of the cuticle of the size of a sixpence being removed by means of a small blister on the back of the neck, as close to the root of the hair as possible, and a similar portion in the hollow beneath, and on the inside of the knee, as the most convenient place: to the wound in the neck, a plate of silver, varying according to the age of the patient, from the size of a sixpence to that of a half-crown, was applied—having affixed to its back part a handle or shank, and to its lower edge, and parallel with the shank, a small staple, to which the conducting wire was fastened. This wire descended the back till it reached a belt of chamois leather, buttoned round the waist—it then followed the course of the belt, to which it was attached, till it arrived opposite the groin on the side it was wished to be used; it then passed down the inside of the thigh, and was fastened to the zinc plate in the same manner as to the silver one. The apparatus so contrived was thus applied:—a small bit of sponge moistened in water, and corresponding in size to the aperture in the neck, was first placed directly upon it—over this a larger piece of sponge of the same size as the metalic plate, also wetted, was laid—and next to this the plate itself, which was secured in its situation by a strip of adhesive plaster passed through the shank on its back, another above, and another below it. If these be properly placed, and the wire which passes down the back be allowed sufficient room that it may not drag, the plate will not be moved from its position by any ordinary motion of the body. The zinc plate was fastened in the same manner—but in place of the second layer of sponge, a bit of muscle answering in size to the zinc plate was interposed: that is, a small bit of moistened sponge being first fitted to the aperture below the knee, the piece of muscle also wetted then followed, and on this the plate of zinc. The apparatus thus arranged will continue in gentle and uninterrupted action from twelve to twenty-four hours, according to circumstances. This last is the longest period that it can be allowed to go unremoved: the sores require cleaning and dressing, and the surface of the zinc becomes covered with a thick oxide, which must be removed to restore its freedom of action; this may be done by scraping or polishing: but it will be better if removed twice a day, both for the greater security of a permanent action, and for the additional comfort of the patient."

